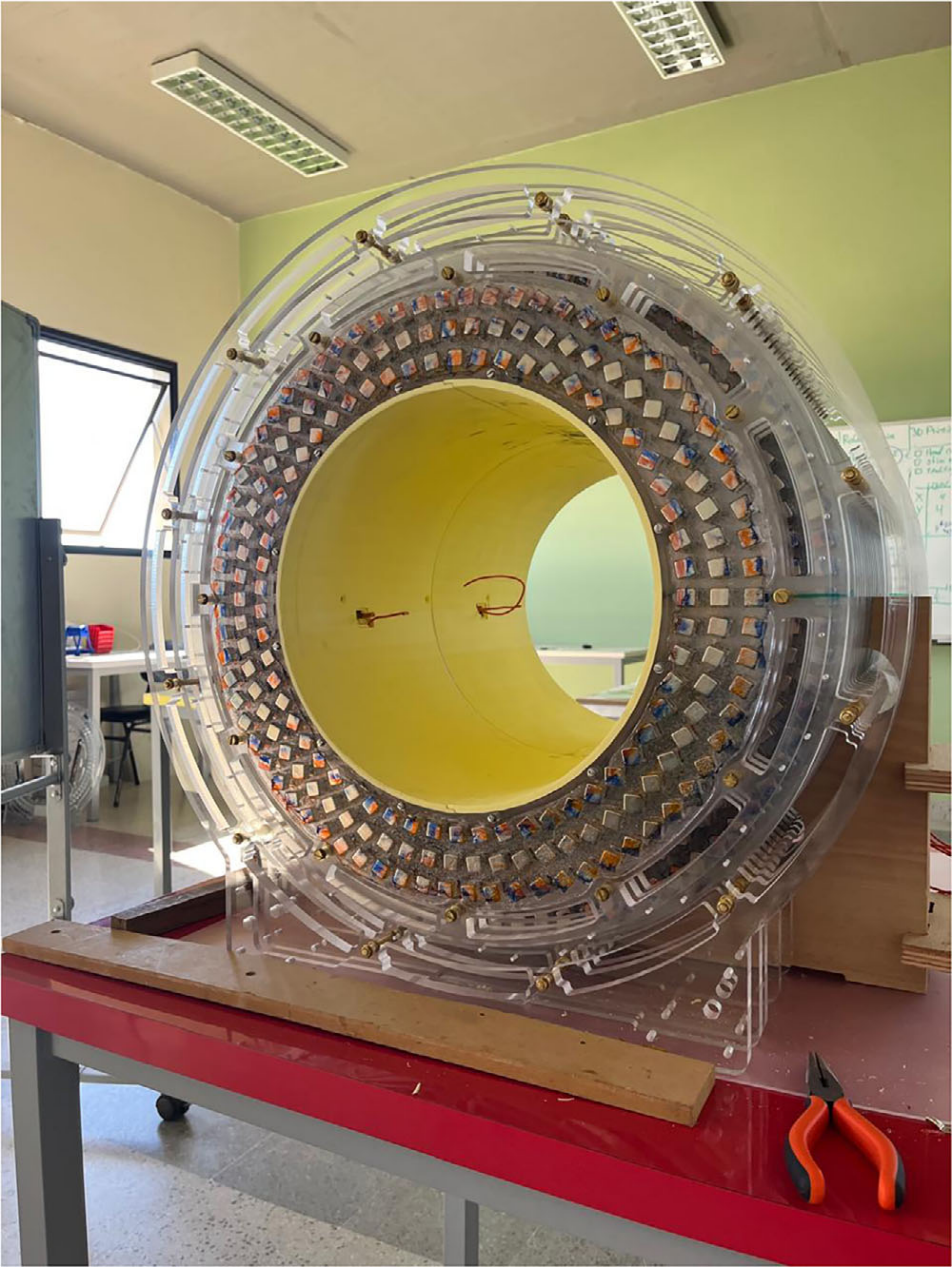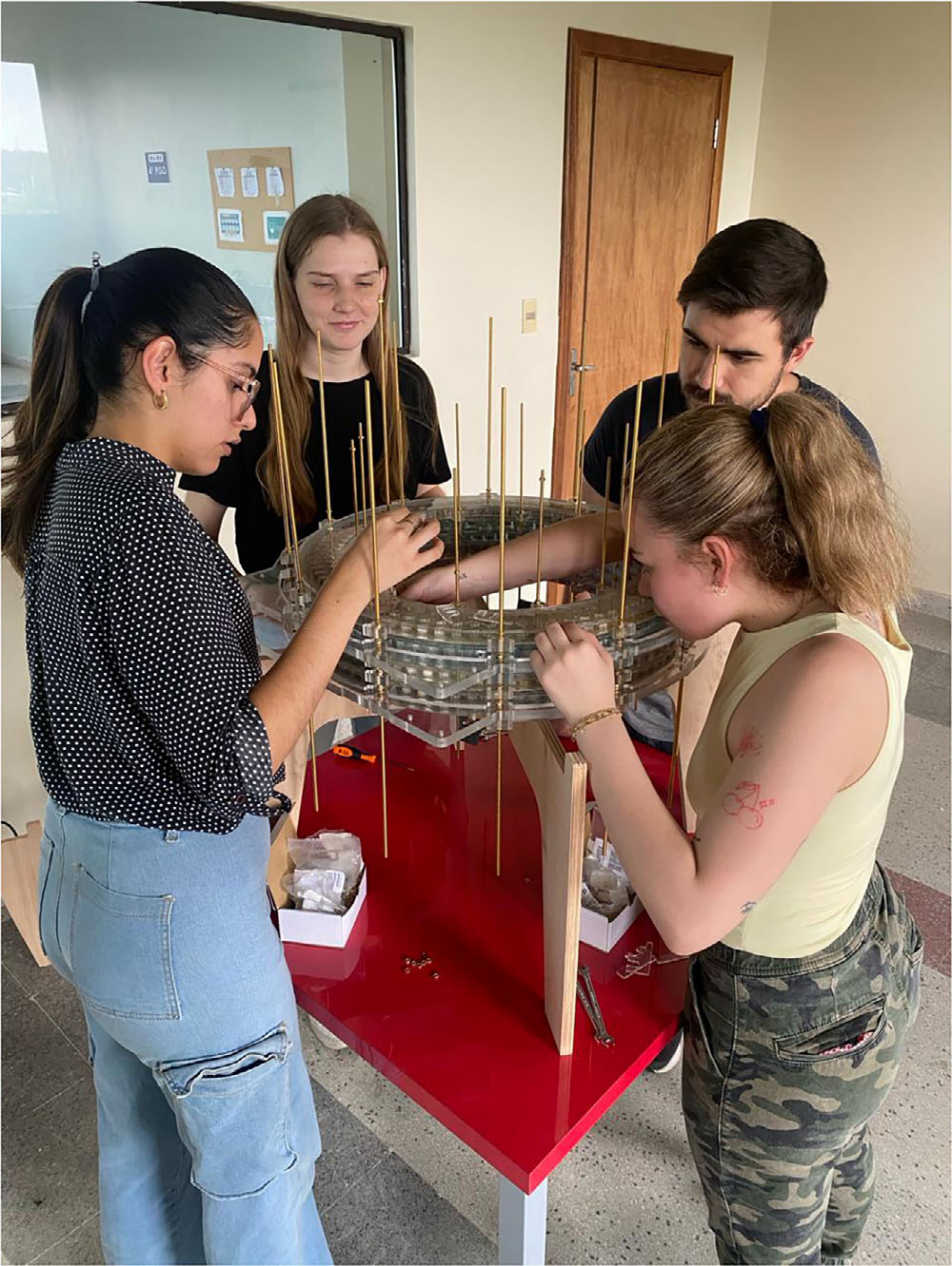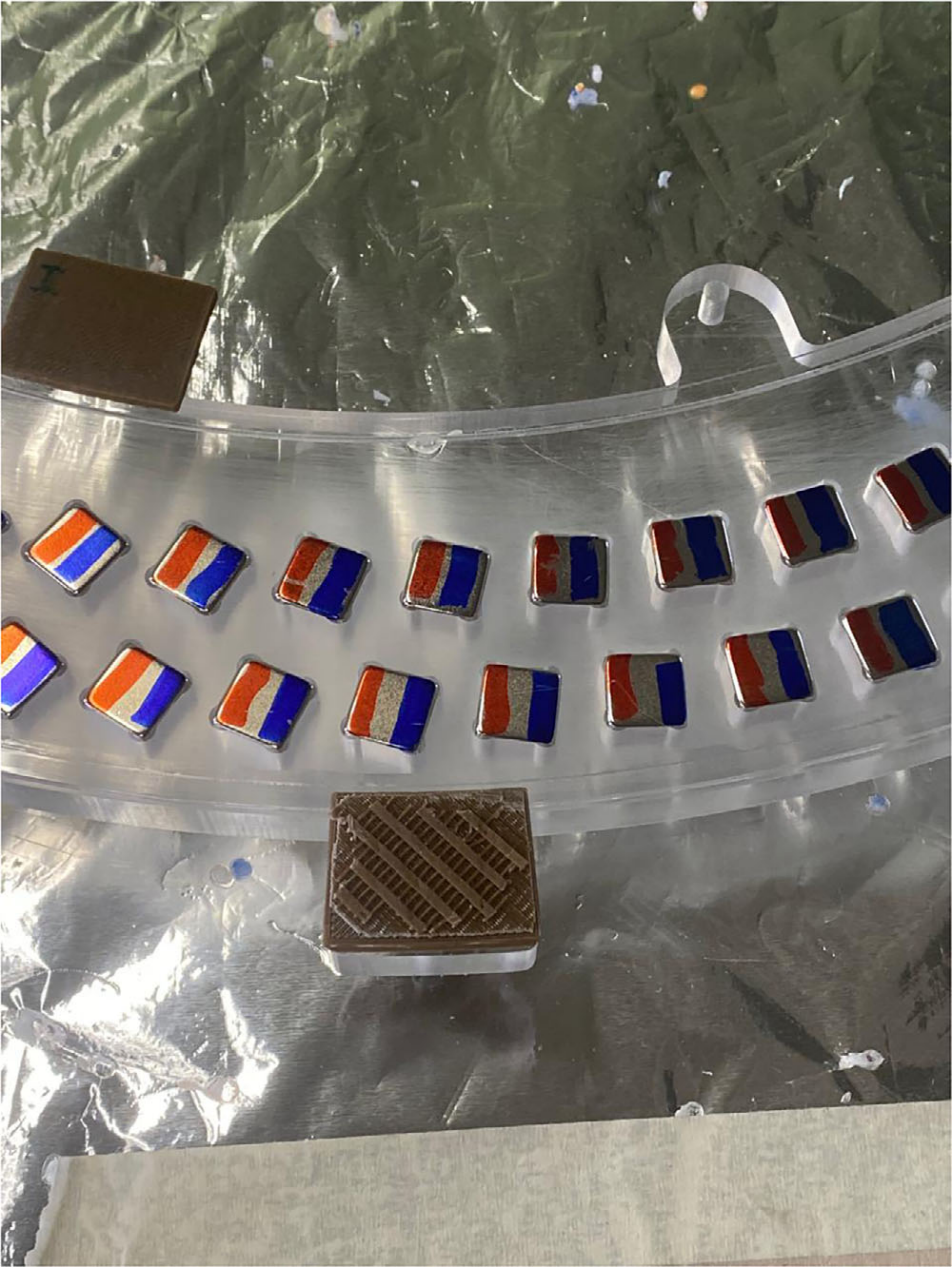# Improving access to global neuroimaging through locally sustained technology

**DOI:** 10.1002/alz.094948

**Published:** 2025-01-09

**Authors:** Joshua Harper, Tavia E Evans, Johnes Obungoloch, Hieab H.H. Adams

**Affiliations:** ^1^ Universidad Comunera, Asuncion, Capital Paraguay; ^2^ Universidad Paraguayo Alemana, San Lorenzo, Central Paraguay; ^3^ Erasmus MC, Netherlands, Rotterdam Netherlands; ^4^ Mbarara University of Science and Technology, Mbarara, Mbarara Uganda; ^5^ Radboud University Medical Center, Nijmegen, Gelderland Netherlands; ^6^ Latin American Brain Health (BrainLat), Universidad Adolfo Ibáñez, Santiago, Santiago Chile

## Abstract

**Background:**

It is estimated that at least two‐thirds of the world lacks access to neuroimaging, including Magnetic Resonance Imaging (MRI). Yet neuroimaging is one of the most informative tools for neuroscience, potentially biasing advances toward populations with access to this technology. This is evident in Alzheimer’s disease (AD) research, where genetics and environment play important roles in disease development and treatment. Recent advances in low field MRI (LFMRI) have demonstrated the utility of this affordable and portable technology, despite lower image quality. Of the existing technologies, the OSII ONE developed by the open‐source imaging initiative is the only device that has been reproduced in low resource settings using primarily local resources. Here we highlight two pilot studies in Uganda and Paraguay to build and sustain OSII ONE devices for neuroimaging studies. We present preliminary results regarding construction, sustainability, and image processing for use in AD and offer suggestions for its inclusion as a tool to increase access to neuroimaging in low‐resource populations.

**Method:**

An OSII ONE system was reproduced in Uganda and Paraguay. In Uganda, all system components were importanted and local human resource guided by international experts was used to complete the build. In Paraguay, only key physical components not available locally were imported and all available local components (including exclusively local human resources) were used for construction.

**Result:**

While the Uganda build was completed more quickly (11 days in Uganda, 6 months in Paraguay), quality was maintained across sites despite difference in personnel and the use of local components in Paraguay. Both projects are maintained locally and current image processing methods could provide adequate quality for brain analysis in AD.

**Conclusion:**

We show the feasibility LFMRI in lower resource settings. Despite the reduction in image quality, valuable structural information can be obtained, extending access to rural populations. This has significant implications for the future of AD imaging in global settings especially considering recent approval of disease modifying therapies that require safety monitoring with MRI. Future work collecting paired LFMRI and conventional MRI with AD patients in South America will further demonstrate the utility of this technology in the field.